# Exploring Men’s Experiences with Follow-Up Care following Primary Treatment for Prostate Cancer in Atlantic Canada: A Qualitative Study

**DOI:** 10.3390/curroncol30120735

**Published:** 2023-11-25

**Authors:** Robin Urquhart, Sarah Scruton, Samantha Radford, Cynthia Kendell, Elias Hirsch

**Affiliations:** 1 Department of Community Health and Epidemiology, Dalhousie University, Halifax, NS B3H 4R2, Canada; 2 Cancer Outcomes Research Program, Nova Scotia Health Authority, Halifax, NS B3H 2Y9, Canada; sarah.scruton@nshealth.ca (S.S.); samantha.radford@nshealth.ca (S.R.); eliashirsch11@gmail.com (E.H.); 3 Department of Surgery, Nova Scotia Health Authority, Halifax, NS B3H 2Y9, Canada; 4 Department of Medicine, Dalhousie University, Halifax, NS B3H 4R2, Canada; cynthia.kendell@nshealth.ca

**Keywords:** follow-up care, prostate cancer, survivorship, qualitative methods

## Abstract

Prostate cancer is a common and life-altering condition among Canadian men, yet little is known about how follow-up care is provided to those who have completed treatment. Despite improving survival rates, survivors experience ongoing needs and are often not provided with support to manage them. This study sought to investigate the post-treatment experiences and needs of prostate cancer survivors and to determine if and how these needs are being met. Using a qualitative description design, prostate cancer survivors who had completed treatment took part in semi-structured interviews. The interviews were recorded and analyzed thematically. The participants experienced varying levels of satisfaction with their follow-up care. While primary care providers played significant roles, continuity of care and specialist involvement varied. Most participants felt unprepared to manage the long-term effects of their cancer due to a lack of information and resources from their healthcare providers. Instead, participants turned to their peers for support. Ongoing physical and psychosocial needs went unmet and had significant impacts on their daily lives. Participants felt that support for these issues should be automatically integrated into their follow-up care. In summary, this study revealed the importance of integrated, patient-centered follow-up care for prostate cancer in Atlantic Canada.

## 1. Introduction

Prostate cancer is the most common cancer diagnosed among Canadian men and the second most common cancer in Canada overall [1]. In fact, one in eight men is expected to be diagnosed with prostate cancer within their lifetime [2]. Due to advances in early diagnosis and treatment, cancer survival continues to improve worldwide. In fact, it is estimated that over 3% of the Canadian population are cancer survivors, and two-thirds of those diagnosed with cancer today will be long-term survivors [3,4]. For prostate cancer specifically, the 1- and 5-year net survival is estimated to be 93% and 90%, respectively [4]. With improved survival, however, comes an increase in the number of individuals who are living with the long-term effects of their cancer and treatment.

Prostate cancer treatment can leave patients dealing with issues such as incontinence, sexual dysfunction, and poor mental health [5,6]. These long-term effects mean that survivors’ medical and supportive care needs after treatment are similar to the level of needs they experienced during treatment, which highlights the importance of comprehensive and well-managed follow-up care [7]. For prostate cancer, follow-up care typically includes routine appointments every three months to one year, with physical exams, prostate-specific antigen (PSA) tests, and other procedures as needed [8].

Currently, there is wide variation in follow-up care practices across Canada [9,10,11,12], and many survivors report poor access to the support and information necessary to manage their ongoing needs [3,13]. Typically, follow-up care has been managed primarily by specialists (i.e., oncologists) at cancer care centers [14,15]. However, due to the increasing number of survivors and decreasing number of specialists resulting from high retirement and burn-out, this model is unsustainable [15,16,17]. Much prior research has shown that alternative models of follow-up care, such as primary care-led follow-up, are equivalent to specialist-led care in terms of safety, effectiveness, cost-effectiveness, and acceptability [18,19,20,21]. In addition, primary care providers (PCPs) are willing to take on the primary management of these patients as long as they have the proper resources and support in place, which could ease the burden on the cancer care system [22,23,24].

Currently, we do not have a good understanding of who provides follow-up care to prostate cancer survivors in Canada and in which settings or survivors’ experiences with care delivery. However, several recent studies in Nova Scotia have found that prostate cancer survivors have more oncologist, surgeon, and primary care provider visits during follow-up than breast, colorectal, and gynecological survivors [25,26,27]. In addition, prostate cancer survivors have been found to report the highest level of needs after treatment compared to breast, colorectal, blood, and melanoma cancer survivors [27]. The purpose of this study was to explore the follow-up care experiences of prostate cancer survivors in Atlantic Canada. We specifically sought to understand survivors’ experiences with follow-up care, their post-treatment needs, and whether and how these needs are met.

## 2. Materials and Methods

### 2.1. Study Design

Guided by qualitative description, we conducted one-on-one, semi-structured interviews with prostate cancer survivors receiving follow-up care in Atlantic Canada (comprised of the Canadian provinces of Nova Scotia, New Brunswick, Prince Edward Island, and Newfoundland and Labrador). As described by Sandelowski, the purpose of the qualitative description is to summarize and describe the informational content of the data with minimal interpretation, although some interpretation is always necessary for researchers to make sense of the data [28,29]. The lead investigator (RU) obtained ethical approval for this study from the Nova Scotia Health Authority Research Ethics Board.

### 2.2. Participant Recruitment

We aimed to recruit 10–12 adult (18+ years) male participants who had completed primary treatment for prostate cancer within the last 5 years and who could speak and understand English. Primary treatment was surgery, radiation therapy, and androgen deprivation therapy (ADT) in the absence of other treatments or active surveillance. We considered active surveillance a form of primary treatment. However, for those on active surveillance, there was no completion; rather, they could continue to be on active surveillance and still participate. Participants could be on ongoing ADT and still take part. Participants were recruited through posters and study summaries shared through social media, hospitals, and organizations and networks that work closely with prostate cancer patients. Interested participants who contacted the research coordinator were invited to take part in a one-hour-long interview. Informed consent was obtained from each participant prior to data collection taking place.

### 2.3. Data Collection

Data were collected from May 2021 to December 2022. Each participant took part in a semi-structured telephone interview carried out by research assistants with experience in qualitative research methods (SR and EH). The interviews took place using videoconferencing software (Zoom for Healthcare). The interview guide was developed based on the study objectives, guided by qualitative interview methods as described by Patton and Rubin and Rubin [30,31]. The open-ended questions sought to understand the survivors’ experiences with follow-up care, their post-treatment needs, and whether and how these needs were met (see Supplementary File S1). Each interview was audio-recorded and transcribed verbatim by an experienced transcriptionist for analysis purposes.

### 2.4. Analysis

Prior to analysis, any identifying information was removed from the transcripts. The interviews were uploaded to NVivo 12 to be analyzed by two research assistants (SS and SR) trained in qualitative research methods under the supervision of an experienced qualitative researcher (RU). To maximize rigor, the first two transcripts were coded and categorized independently by each research assistant into broad conceptual categories. Coding was primarily inductive, with no a priori codes. The researchers then met to compare the coding, ensure it represented the data, and develop a preliminary coding scheme to guide the rest of the analysis. The remaining transcripts were split evenly between the two research assistants and analyzed based on the coding scheme. One research assistant (SS) then went through the conceptual categories to collapse, review, and refine the main themes, along with relevant quotes to illustrate the key concepts. These analyses were then shared with RU, who independently reviewed all coding and associated decisions and assisted with the collapsing of codes into sub-themes and themes and refinement of final themes.

## 3. Results

**Figure 1:**
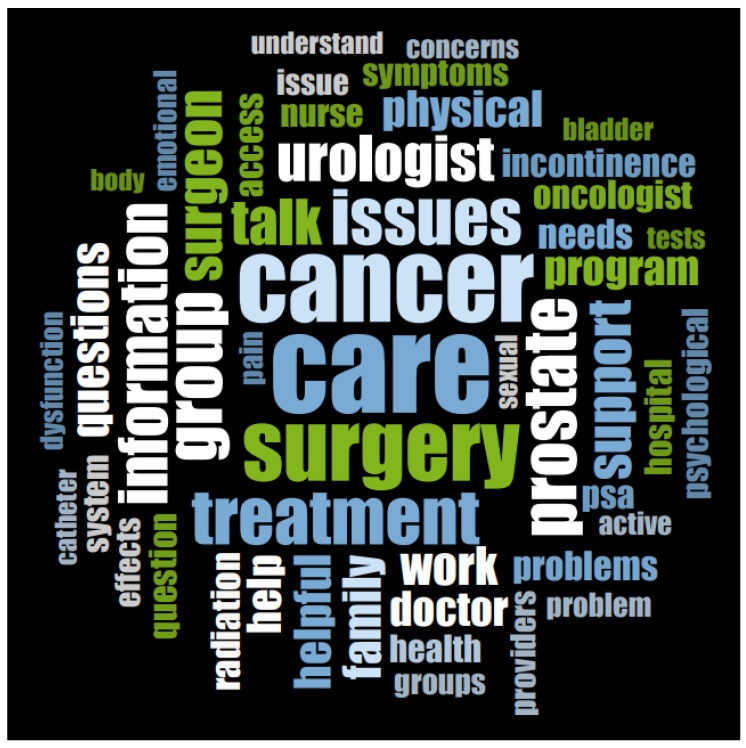
Word cloud of common concepts present in the dataset. Larger words depict a concept that occurs at greater frequency.

A total of eight prostate cancer patients were eligible for this study and took part in interviews. Primary treatment involved surgery (n = 6), radiation therapy (n = 1), and active surveillance (n = 1). All participants resided in urban or suburban areas. Seven were retired at the time of participation. Participants reported having appointments or tests every 3 months to annually, depending on their level of need and how far they were from treatment. Every survivor reported receiving regular PSA tests (i.e., every 6 months). Other tests they received included MRIs, biopsies, ultrasounds, and bladder tests. Follow-up care was provided by and shared between a variety of providers, including specialists (i.e., urologists, surgeons), family doctors, nurses, and physiotherapists. The final four themes involved physician roles and continuity, feelings of unpreparedness, ongoing physical needs and their impact on recovery, and psychosocial concerns and lack of support. Table 1 depicts these main themes alongside sub-themes. Figure 1 provides a word cloud of the common concepts that were present in the dataset. 

### 3.1. Physician Roles and Continuity during Follow-Up Care Impact the Experience

As participants described their experiences with follow-up care, it was clear that there were key differences that impacted their satisfaction with the care they received. Most commonly, their satisfaction is related to their physicians’ involvement (or lack thereof) in their care and to suboptimal coordination and continuity of care across physicians. For the most part, participants were satisfied with the number of appointments and tests they received, with the exception of some who expressed concerns about delays due to COVID-19 or issues booking appointments. Most (six of eight) participants felt their PCP was an integral part of their follow-up; in fact, approximately half deemed their PCP was in charge of their post-treatment care. Many participants expressed surprise by how little their specialist was engaged in this period of care.

“My GP’s really good. He does a PSA every six months for me. So that kind of keeps…you know, gives you some information on where the prostate’s going or what’s [the] situation with the prostate”.

[P6]

“I was surprised how uninvolved my urologist was. Really, you know, after I’d had the surgery, there was nothing mentioned. I didn’t get to see him again. I thought at the time that I would be way more involved with my urologist… No follow-up, no nothing. He was fine going in. Okay, he didn’t have a lot of time. He told me about this, that and the other. But coming out of the surgery, I thought that my urologist would be more important. Not so”.

[P2]

Some participants expressed concerns about continuity of care regarding the inconsistency in which they were seeing the same physicians. Some felt there were too many physicians in their care, and others grappled with finding new physicians after losing theirs to retirement.

“Well, yeah, my regular family doctor retired like immediately after the surgery. So I ended up without a family doctor for… I think I was without for about eight months. And then I finally found a GP, and he was great. However, he decided to go to [City] to re-specialize. So I ended up without a family doctor again”.

[P2]

Though there were no concerns about the competence of new physicians, many participants reported a preference to stick with someone who had a good understanding of their case and personal history. Overall, the lack of continuity of care negatively impacted their satisfaction with care after treatment.

“So I’ve had a succession of people. And that in itself has been kind of unsatisfactory. I mean they were all competent, I think. But it’s a chain. I would have preferred, of course, to stay with one person throughout”.

[P1]

There were mixed experiences in terms of the quality of the relationship between participants and their care providers. Half voiced appreciation for the compassion and empathy that their physicians provided, and they were grateful to them for patiently listening to their concerns and providing them with support in return. They were also grateful that they felt they could reach out at any time to ask questions or express concerns rather than waiting for their routine appointments.

“I think every time I’ve met with them, they’ve been patient--no pun intended. They answered any questions I had. They didn’t rush. There was no sense of, well, we’ve got to get you out to get someone else in. And I know how busy they are. I never felt that. I never felt they were reluctant to answer any of the questions that I had”.

[P7]

The other half, however, described their physicians as being neither empathetic nor patient. Although they were confident in the medical care they were receiving, they felt their concerns (e.g., related to long-term side effects) were brushed aside, or they felt too rushed to ask any questions.

“Yeah. And in a lot of cases, if I did ask… Over the years, if I asked my oncologist some questions I wanted the answers, I was made to feel like, ‘I’m too busy. I don’t have time. I don’t have time to answer your questions. You just depend on me for your answers. Like I’m God here, and I’ll tell you when and what needs to be done, and you just show up’”.

[P4]

### 3.2. Feeling Unprepared for the Post-Treatment Period

Following treatment, most participants felt they did not have the information they needed to manage the long-term side effects resulting from their cancer and treatment, and they described limited knowledge of how to get back to “normal”. Nearly all were frustrated that they had not received education from their healthcare providers, and they were often unaware of the resources available to manage their ongoing issues. This came as a surprise, as they felt this type of information should be coordinated as an integral part of their follow-up care. As a result, participants described feeling left on their own to seek informational support.

“If they don’t even contact you to see how you’re doing, how in the hell are you going to get anything, right? And of course, I don’t know what’s out there… You know, just nothing. You know, like when you’re in [the clinic], the most you’re going to learn is you see that pamphlet over there, that has to do with prostate cancer. Go read that, see if you can find something in that that’ll help you”.

[P4]

“The biggest problem when you go to the doctor’s office or the surgeon’s office or any office of a specialist, they don’t give you information to try to help you get through this. And I had to do that on my own”.

[P6]

Most commonly, information was sought from peers by joining support groups. These were described as an excellent source of information as they trusted the advice that came from those with lived experiences. They also felt that this type of support made it easier to ask questions about sensitive topics as they knew their peers had gone through similar experiences. They described receiving treatment advice, tips and tricks for managing side effects, where to find other resources, practical support, and emotional support. The participants felt they would not have learned about those resources or information without their peers. They also appreciated that experts often joined support groups to educate survivors on how to safely manage their ongoing needs at home.

“And I do find that it is a benefit because they have had speakers on, you know, who have expertise in dealing with prostate cancer and cancer generally. So that has been very helpful because you can ask questions and get information. And they’ve also had, although the pandemic has altered it a fair bit, but actual sessions where you could go, and there was someone who is an expert in the field of… a radiation oncologist in September speak to the group. So I found that quite helpful, quite good”.

[P8]

Outside of support groups, all but one participant used the Internet to meet informational needs. This included using search engines to address their questions, watching YouTube videos, signing up for e-mail lists, or purchasing self-help/rehabilitation programs.

“I do a lot of research on anything that’s wrong with me here that I’m affected with. And that’s where I got the bulk of my information, was from correspondence and videos—especially a lot of YouTube videos, which discuss things prior to surgery, like nerve sparing, non-nerve sparing, what to expect afterwards, penile rehabilitation—which I do on my own”.

[P4]

### 3.3. Ongoing Physical Needs Impact Optimal Recovery and a Return to Normal

All participants reported experiencing sexual dysfunction and/or urinary incontinence as a result of their treatment, both of which persisted despite attempts to manage them (i.e., surgery, rehabilitation, medication). Though some were supported by healthcare providers who offered a variety of resources to manage these symptoms, most felt they were on their own to find solutions.

“I’m pretty much left on my own to figure things out. You know, the two main difficulties with a prostatectomy are urinary incontinence and erectile dysfunction. And there hasn’t been any attention or, you know, anybody told me what or how I should deal with those problems”.

[P5]

Participants felt that ideal follow-up care should include automatic referrals to support for these issues following diagnosis so they are not forced to seek help themselves. The suggested solutions ranged from these supports becoming a standard of care to something as simple as providing a list of services and providers who can be contacted that are comfortable managing prostate cancer issues (i.e., psychologists, navigators, physiotherapists). Those who had access to these types of support acknowledged that they were important aspects of their recovery.

“I think it would have been nice if it had been suggested to me within the context of the system that if I was having concerns about erectile dysfunction, that here, there’s somebody that you could see within the system. Now, I have spoken with somebody, someone outside the system. But I didn’t find that very helpful. But within the system, no, nobody has handed me someone’s card and said, ‘You should talk to this person to deal with how you should cope with this handle this, or remediate some of your trepidation around it’”.

[P7]

Participants described these ongoing needs as having a large impact on their everyday lives and hindering their ability to return to normal. For those with sexual dysfunction, they described negative impacts on relationships, low self-esteem, and feelings of defeat. Ongoing incontinence seemed to be more successfully managed, but those who experienced it described feeling embarrassed and frustrated. Other physical side effects of their treatment that impacted the ability to return to normal included fatigue, issues regulating body temperature, and weight gain.

“I mean no doubt that I’m not a young man, but I’m not old either. But that was probably more on my mind. So at the beginning, yeah, it was a very… it was a hard struggle. Which people just don’t understand because you lose your man… you kind of lose the manhood. You know what I mean? And still at times it bothers me that, you know, the performance of it is not there. But, you know, a lot of people say, “Well, [participant], you’re up in age now. So, you know, it’s a good thing that it happened now and didn’t happen earlier”. … So I can understand that, too. But it is troublesome for the mind. You know, the mind works quite a bit on that. But you have to just say, ‘well, listen, there’s a lot worse out there, you know’”.

[P6]

### 3.4. Psychosocial Concerns Are Prevalent, yet Supports Are Lacking during Follow-Up Care

All but one participant described dealing with ongoing and substantial psychological issues as a result of their cancer and treatment. These were mostly related to sexual dysfunction, which impacted their self-esteem and relationships, as described above. This resulted in feelings of depression, anxiety, or overall distress.

“I did battle with [erectile dysfunction] a lot. I actually, I struggled with depression. I started drinking more. I had a really hard time with depression after my prostate was removed. Not right away but within a couple of months afterwards, my depression got overwhelming. And I was self-medicating”.

[P3]

“I mean I think you can chalk [emotional or psychosocial issues] up to both the erectile dysfunction… I mean there’s a huge level of sadness over that. And not only for myself, but also my wife”.

[P7]

Some of the participants described seeking support for their psychosocial issues as challenging due to a certain degree of stigma and embarrassment around discussing these sensitive topics. In addition, most participants stated that their mental health was never addressed by their physicians, who were described as being unprepared to deal with this type of issue.

“Certainly, like the general question was, you know, why do I feel this way? Why is my marriage suffering? Why? I think if I, if I experienced any of that, I guess either my GP or my urologist should have been able to refer me. But nobody did a mental health check. And I think that’s important. Certainly listening to the guys and wives in my support group, mental health is one thing that just gets sidelined”.

[P2]

Participants emphasized that psychosocial support is necessary for their recovery and, therefore, should be automatically integrated into their care as soon as they are diagnosed. However, many remained unaware of any health system resources available to help them and felt they were left to find support or attempt to manage their psychosocial needs on their own.

“I’ve said before, the one thing that I would like to see changed is being able to see a mental health professional before… like straight after diagnosis because it screws with your brain. And then afterwards to explain, you know, the feelings that you’re going to have during recovery”.

[P2]

Participants described several activities and resources that helped them manage their psychosocial needs after treatment. First, although resuming normal routines and activities (i.e., hobbies, work, exercise) was challenging due to ongoing side effects, including fatigue, participants felt the normalcy that comes with returning to these activities helped with their psychosocial recovery.

“I did go back to work. And I find that when I’m there and when I’m in doing my ground work, I find I feel a lot better. You know what I mean? Like everything is clear. My mind’s clear. I like it. And so, you know, no, I think that’s very important for anybody to keep active, I guess”.

[P6]

Second, peer support (often accessed in the form of support groups) was an extremely important resource for emotional needs. Participants said it was easier to open up emotionally to peers who had similar experiences, and the support they received in return was imperative.

“And so he put me onto the local [City] group. Which was an absolute godsend. And I frequently tell people, I say the surgeon and his team fixed my body, but it was the support group that fixed my head. Because tackling cancer, it’s not just about what’s growing and then taken out of your body, it’s how cancer really screws with your brain. When you’ve got that hanging over you, you cannot think logically. It really does mess with your head”.

[P2]

Those who chose not to access support groups described concerns about their usefulness, misinformation, stigma, or geographical barriers. The majority of the participants described the importance of having family support for emotional and practical needs, though many were concerned about how the burden of their cancer might impact their loved ones.

“So that has an impact on me because I worry about how it would affect her emotionally. And sometimes I feel like I’d like to tell her. And then other times I feel like, no, it’s better just not to say anything because the emotional fallout would be too much”.

(P8)

In all, participants described how psychosocial support encouraged them to take action to improve their quality of life and gave them the tools to do so.

## 4. Discussion

This study sought to explore prostate cancer survivors’ experiences with follow-up care, their post-treatment needs, and whether and how these needs are met. In summary, participants described varied experiences in terms of receipt of follow-up care, impacting their satisfaction with their care. Nearly all felt their PCP was involved in their follow-up care, and around half felt that they were primarily responsible for it. Poor continuity of follow-up providers led to dissatisfaction for many participants. Most felt they did not receive the informational and emotional support required to safely manage the long-term effects of their cancer and treatment. They felt these supports should be something that is integrated into their care rather than something they need to seek out themselves. This was especially important as the majority of participants were dealing with several unmet physical and psychosocial needs due to their cancer and treatment (i.e., erectile dysfunction, distress, urinary incontinence). As a result, the participants turned to their peers for information and emotional support. Support groups were continually referenced as an extremely important resource as the participants valued the support that comes from those who have gone through similar experiences, such as dealing with sexual dysfunction.

Little is known regarding how follow-up care is provided to prostate cancer patients within Canada, and we have a limited understanding of the processes and outcomes that are important to the patients themselves for them to feel satisfied with their care. In the current study, variations in follow-up care were described in relation to continuity of care. Some participants described a lack of continuity due to seeing a number of different providers, and some faced the loss of physicians due to retirement. Though not described in the current study, this lack of continuity can cause concerns as cancer survivors may be unaware of who is responsible for certain aspects of their care and, therefore, who to reach out to if issues arise [32]. In addition, poor continuity of care has been found to negatively impact patient quality of life and may result in over- or misuse of the healthcare system [33,34]. Though nearly all participants in our study stated that both their specialists and PCPs were involved in their follow-up care in some capacity, around half felt their PCP was in charge, and they were surprised and confused by the lack of involvement of their specialist. It is not uncommon for survivors to feel surprised by the lack of contact with their specialist following treatment, and it may result in them feeling abandoned by the healthcare system [10]. This is heightened by the fact that many survivors, including those in our study, are not given adequate information or resources to manage their long-term side effects before this transition occurs [10]. This lack of information and sudden transition may explain why other research has found patients would prefer if their specialist stayed more involved in their follow-up care or if their PCP was more involved during the treatment phase of cancer [35,36]. If these transitions are well-managed with appropriate resources and support in place, patients may be more satisfied as their needs are more likely to be met. Exploring ways to incorporate psychosocial education and support into the standard of care and how to optimally support transitions in care for prostate cancer survivors are two areas of future inquiry.

Interestingly, participants in this study were not overly concerned by who was in charge of their care as long as they were given the appropriate support and resources to meet their needs. This finding has important implications, as the current model of follow-up care, which is typically led by oncologists, is unsustainable. The growing number of cancer survivors in Canada is accompanied by a growing physician shortage [16]. Cancer specialists are reporting very high levels of exhaustion, stress, burnout, and depersonalization, which is leading many to consider a career change [17]. This highlights the importance of implementing alternative follow-up care pathways, such as those led by primary care providers, in order to ease the burden on specialist care. Though participants in this study felt comfortable being seen by someone other than their oncologist, they emphasized that their care would have been improved if they had been provided the necessary resources to effectively manage their late and long-term effects. Instead, they were left to seek these on their own. This is a common issue within survivorship care, with many survivors leaving treatment feeling unprepared for their post-treatment recovery [10]. A national Canadian survey found that the large majority of cancer patients experience unmet needs after treatment, many of whom found it difficult to access the support and resources needed to overcome the late and long-term effects of cancer [37]. It was also found that cancer survivors who do not seek support for these issues often do not want to ask for support, are not aware of the services that exist to help them, or they feel that nothing can be done to help them [37]. This is compounded by the fact that survivors often feel they do not receive as much support from their physicians compared to when they were in treatment [10,38]. As described by our participants and corroborated by existing literature, survivors sometimes feel rushed by their physicians during follow-up, and a lack of compassion and empathy can mean they do not feel comfortable asking questions [32,39]. One potential solution suggested by the participants in our study was that psychosocial resources and education become integrated as a standard of care. Some intervention studies have tested automatic screening for outcomes such as depression or distress followed by suggestions or referrals for support as needed, with positive outcomes [40,41,42]. In addition, some federal or national cancer organizations/unions call for rehabilitation services to be offered to all cancer patients [9]. However, in general, referrals to psychosocial support appear not to be automatic in most settings and are lower than they should be due to healthcare provider knowledge of and attitudes toward this type of support [43]. Programs do exist in Canada that were developed to meet the needs described by participants in this study (for example, see pcscprogram.ca (accessed on 24 November 2023) and pcpep.org (accessed on 24 November 2023)). Understanding how these programs work and their impacts can enable scale-up more broadly to support survivors during and after completing primary treatment.

As discussed by participants in this study, many survivors turn to their peers in the absence of support from their healthcare providers. This is a common theme within the cancer literature; peers offer a unique type of support as they can understand what one another has gone through and offer advice or recommendations based on personal experience [44,45]. This support reduces isolation, improves quality of life and cancer-related knowledge, and provides cancer patients and survivors with a sense of control over their cancer [44,45,46,47,48]. With that said, peer support may work best when offered in conjunction with professional support, as there may be concerns about misinformation [49,50]. Because of this, it is important that survivors are able to leverage both formal and informal resources so they have well-rounded support to meet their needs. Testing ways to sustainably deliver peer support to the large number of survivors who would benefit from these services should be a focus of future study.

### Limitations

This study captured data on the experiences of prostate cancer survivors in one region of Canada. Therefore, the findings may not represent follow-up care experiences in other Canadian jurisdictions and beyond. Participants may also not be representative of the broader prostate cancer population, given our recruitment strategies were non-random and included the distribution of posters and study information through clinical and community organizations and social media. Moreover, recruitment was challenging, and although we sought to recruit 10–12 participants, only eight participants ultimately took part in the study, a relatively small sample size. One perceived reason for this challenge was the global COVID-19 pandemic, which decreased recruitment opportunities as much care shifted to virtual means. Another potential limitation is that participants’ follow-up experiences may have been influenced by pandemic-related health system disruptions. Unfortunately, there is no way to delineate the impact of these disruptions from care received outside of the pandemic. Nonetheless, the findings align with researchers’ findings elsewhere and prior to the pandemic for prostate cancer as well as other cancer types. This suggests the findings are transferable across settings and time periods. The findings provide a guide to begin improving follow-up care for prostate cancer survivors across Atlantic Canada, particularly in the context of prior findings that prostate cancer survivors have higher healthcare utilization and higher unmet needs than survivors of other prevalent cancers (e.g., breast and colorectal cancer) [25,26,27].

## 5. Conclusions

In conclusion, this study identified that prostate cancer patients’ follow-up care experiences are varied, yet most have ongoing needs that are often not met by the health system. Issues identified by the participants included a lack of continuity of care and a lack of informational and psychosocial support from their care providers. Participants were often not provided the support and resources required to meet their needs and, therefore, turned to their peers for support. They also discussed the emotional toll their diagnoses and treatments have on their partners. The participants felt that resources such as psychological support and patient education should be integrated as a standard of care. All of these issues require future study. Understanding how existing programs and services work, including their core components, would help others implement and scale such programs in their jurisdictions.

## Figures and Tables

**Table 1:** Main themes and sub-themes.

Main Theme	Sub-Themes
Physician roles and continuity during follow-up care impact the experience	Follow-up care experiences vary
	Most consider their family physician integral to their follow-up care
	Involvement and availability of care provider
	Quality of relationship with provider
	Suboptimal coordination and continuity of care
Feeling unprepared for the post-treatment period	High informational needs during follow-up
	Role of peers and Internet to meet informational needs
Ongoing physical needs impact optimal recovery and a return to normal	High needs regarding sexual dysfunction and urinary incontinence
	Needs often go unmet or persist
	Ongoing needs have a large impact on everyday life
	Finding support for these needs is important to support recovery
Psychosocial concerns are prevalent, yet support is lacking during follow-up care	High and ongoing psychosocial needs
	Psychosocial needs are not met by the care team
	Peer support is key for psychosocial needs/health
	Need for automatic referrals after treatment
	Family support is important but may negatively impact loved ones

## Data Availability

The data presented in this study are available on request from the corresponding author. The data are not publicly available due to privacy and confidentiality considerations.

## Supplementary Materials

The following supporting information can be downloaded at: https://www.mdpi.com/article/10.3390/curroncol30120735/s1. File S1: Interview guide.
