# Supplementary material for: Exploring Men’s Experiences with Follow-Up Care following Primary Treatment for Prostate Cancer in Atlantic Canada: A Qualitative Study
Source: Curr Oncol. 2023 Nov 25;30(12):10111–23. doi: 10.3390/curroncol30120735 (PMC10742932; doi:10.3390/curroncol30120735)
Supplement: Supplementary file 1 [file curroncol-30-00735-s001.zip › curroncol-2683544-supplementary.pdf]

Interview Guide

Note: Prior to beginning the interview, reiterate to participants that this study is focused on care received *after* the completion of primary treatment for prostate cancer. Confirm primary treatment type with participant.

<u>Experiences with follow-up care</u>

- Tell me about the care you have received since completing your primary cancer treatment.
    - Which provider(s) do you see?
    - How frequently?
    - What tests do you receive?

- What are the reasons that you receive care from that provider/combination of providers?

- How would you describe the care you have received since completing primary treatment?

- What has been positive about the follow-up care you have received? Negative or challenging?

<u>Whether and how post-treatment needs are met</u>

- Since completing your treatment, what issues have you experienced?
    - *Physical* symptoms (e.g., fatigue or tiredness, changes in sexual function, changes to concentration/memory, bladder issues, pain)?
    - *Emotional/psychosocial* issues (e.g, anxiety/stress, depression/sadness, changes in sexual intimacy, body image issues, relationship issues with family/partner or friends/colleagues)?
    - *Practical* issues? (e.g., returning to work, getting to and from appointments, paying for costs associated with health care)?

- Have these concern/issues been addressed by your care providers?
    - If so, how?
    - If not, why?

- Since completing your treatment, are there are specific needs that you have experienced that you have not been able to address?

- Since completing treatment, have you sought out access to any *informational* resources (e.g., information about the side effects of treatment, or information about recurrence)?
    - Were you successful in finding the information you needed?
    - Where did you find it?
    - Was it easy to find?
    - Was it helpful? How so/why not?

Factors to consider in designing strategies to improve follow-up care.

- Think about the best possible care that you could receive after completing primary treatment. What does this look like for you?

- Thinking about the care that you have received since you completed primary treatment, what things have made it easier/harder to access the care you need to address your needs and concerns?

- Are there any specific factors related to your own personal situation that have positively or negatively impacted your ability to access care to address your needs (e.g., travel, costs, pain or other physical symptoms, support from family/friends)?

- Based on your experiences, what could be done to improve the overall quality of care provided to prostate cancer patients once they have completed primary treatment?
